# Dental and Medical Service Utilisation in a German Population – Findings of the LIFE-Adult-Study

**DOI:** 10.3290/j.ohpd.a45412

**Published:** 2020-10-26

**Authors:** Jana Schmidt, Dirk Ziebolz, Samira Zeynalova, Markus Löffler, Katarina Stengler, Kerstin Wirkner, Rainer Haak

**Affiliations:** a Dental Assistant/ Researcher, Department of Cariology, Endodontology and Periodontology, University of Leipzig, Leipzig, Germany. Data analysis; wrote the paper.; b Assistant Professor, Department of Cariology, Endodontology and Periodontology, University of Leipzig, Leipzig, Germany. Data analysis; wrote the paper.; c Statistician, Institute for Medical Informatics, Statistics, and Epidemiology, University of Leipzig, Leipzig, Germany; LIFE Leipzig Research Center for Civilization Diseases, University of Leipzig, Leipzig, Germany. Conceived and designed the experiments; data analysis; contributed reagents/materials/analysis tools; wrote the paper.; d Professor and Head, Institute for Medical Informatics, Statistics, and Epidemiology, University of Leipzig, Leipzig, Germany; LIFE Leipzig Research Center for Civilization Diseases, University of Leipzig, Leipzig, Germany. Conceived and designed the experiments; contributed reagents/materials/analysis tools.; e Assistant Professor, Clinic for Psychiatry, Psychosomatics and Psychotherapy, Helios Park Clinic Leipzig, Leipzig, Germany. Conceived and designed the experiments; contributed reagents/materials/analysis tools.; f Assistant Professor, LIFE Leipzig Research Center for Civilization Diseases, University of Leipzig, Leipzig, Germany. Conceived, designed and performed the experiments; contributed reagents/materials/analysis tools.; g Professor and Head, Department of Cariology, Endodontology and Periodontology, University of Leipzig, Leipzig, Germany. Conceived and designed the experiments; wrote the paper.

**Keywords:** dental attendance, prevention, high-risk groups, cross-sectional population-based study, high-risk strategy

## Abstract

**Objectives:**

This study investigated utilisation behaviour of the dentist compared to general practitioners (GP) and medical specialists in a German cohort under consideration of risk indicators for irregular dental attendance.

**Methods:**

Analysis of the results of the population-based LIFE-Adult-Study (Leipzig, Germany) was performed. A total of 2231 participants of the LIFE-Adult-Study were randomly selected to complete the relevant questionnaire, considering medical attendance behaviour. Associations of self-reported medical conditions, including dentaland medical attendance, sociodemographic factors, as well as self-reported general health status and oral health complaints were determined.

**Results:**

Of the 2231 participants who were included in the analysis, 14.2% reported not to have visited the dentist during the preceding 12 months. There could be shown a more selective utilisation behaviour towards medical services in smokers, men, low socioeconomic status and depression. Women were more likely to attend the dentist than men (OR = 1.8, CI = 1.4–2.3). Smoking (OR = 0.7, CI = 0.6–1.0), low socioeconomic status (OR = 0.6, CI = 0.4–0.8) and depression (OR = 0.6, CI = 0.4–0.9) were related to less dental attendance. Additionally, persons who do not visit the dentist regularly showed less attendance of the GP as well as medical specialists (p <0.05). Depression could be shown to be an additional risk factor for unfavourable utilisation behaviour towards the dentist.

**Conclusions:**

The results showed differences in dental and medical attendance, depending on different patient-related factors. Focusing attention towards high-risk groups might improve dental as well as medical utilisation behaviour, and therefore health status as well.

A person’s oral health has been shown to influence general health status, subjective well-being and social acceptance.^8^,^15^,^16^ Therefore, it plays a crucial role for health as a multidimensional construct, as defined by the World Health Organization.^26^Dental issues are often less regarded in the wider health discourse.^3^ However, caries and periodontitis are the main chronic oral diseases cumulating during the life course and are largely irreversible.^11^ Among other socioenvironmental factors determining oral health, it could be shown that individual, professional and community measures are effective in preventing most oral diseases.^15^,^17^ Consequently, for the maintenance and/or establishment of oral health, routine dental attendance is of great importance and should be performed regularly, adopted for the individual risk of a patient.^2^,^19^,^23^

The current population-based survey of German inhabitants, the Fifth German Oral Health Study (DMS V)^12^ identified statistically significant differences in younger adults, aged 35 to 44 years, with 72% visiting the dentist regularly, whereas 28% showed symptom-based dental attendance.^11^ In the younger senior citizen, aged 65 to 74 years, 89.6% showed regular dental attendance and only 10.4% utilised dental services symptomatically. Overall, dental utilisation in the German population can be considered control-orientated.

In literature, different models dealing with the influence of life course on oral health-related behaviour are described.^3^,^23^ Socioeconomic status (SES) was shown to have a strong influence on dental attendance.^3^,^11^ Additionally, an association to age, gender and smoking status was found in former studies as well.^5^,^23^

However, there are still patients showing unfavourable dental utilisation behaviour and more caries and periodontitis compared to the vast majority of people.^11^ They often share lifestyle-related and preventable risk factors, eg, smoking, with widespread systemic diseases, as cardiovascular events (CVE) and diabetes mellitus (DM).^4^,^9^,^22^ Therefore, they represent a target group for high-risk strategy in prevention. In this context, it is reasonable to elucidate the association between attendance of the dentist in comparison to the general practitioner (GP) and medical specialists.

The aim of the current study, dealing with data of the population-based cross-sectional LIFE-Adult-Study, was to analyse dental care utilisation in comparison to medical attendance, differentiating between the GP and specialists. Beneath socioeconomic and epidemiologic factors, the presence of general diseases (eg, DM, CVE) and high-risk factors (eg, smoking, low SES) were taken into account. It was assumed that general diseases (DM, CVE and depression) present risk factors for an unfavourable utilisation behaviour towards the dentist but not the GP or medical specialists.

## METHODS

### Survey Design

The investigation was performed in nearly 10,000 study participants aged 18 to 79 years of a cohort collected from the population of Leipzig, an Eastern German city with 560,000 inhabitants.^21^ The LIFE-Adult-Study aims at monitoring the development of diseases. Patient recruitment and response rate are described in detail elsewhere.^14^ Data on socioeconomic and sociodemographic factors were obtained in a structured interview. Information about medical, including dental, attendance, were obtained in participants aged 18 to 79 years. The questionnaire was applied randomly in a representative cohort considering all age groups.

The baseline examination was conducted from August 2011 to November 2014. The participants completed a detailed study programme comprising medical assessments and questionnaire surveys.

The study was designed in accordance with the Declaration of Helsinki and with approval of the Ethics Committee of the University of Leipzig (Reg. No. 264-10-19042010).^27^

### Measures

Based upon the data of the LIFE-Adult-Study information, considering (a) age (age groups: 18–34, 35–44, 45–54, 55–64, 65–74, 75–79); (b) gender (male and female); (c) socioeconomic status (SES, classified in high, moderate and low^25^; (d) smoking (yes: current smokers, no: non-smokers, including former smokers); (e) cardiovascular event (CVE, yes or no); (f) diabetes mellitus (yes or no); (g) depression (yes or no); (h) visit to the dentist during the last 12 months (yes or no); (i) visit to the GP and, differentiated: (j) visit to a medical specialist (gynecologist, psychiatrist, orthopaedist, ENT physician, dermatologist, cardiologist, ophthalmologist, neurologist, radiologist, urologist) during the last 12 months (yes or no); (k) subjective oral health complaints (pain at the teeth, pain at the gingiva, inflammation of the oral mucosa) during the last 4 weeks (yes or no), were obtained.

### Analysis

In the first step, descriptive statistics for analysing the study population’s demographic features and univariate analyses were performed. Chi-squared and, if necessary, Fischer’s exact tests were applied to determine the association between medical attendance (dentist and other physicians) and sociodemographic, behavioural and medical factors and for identification of factors influencing dental attendance behaviour. Furthermore, the association between subjective oral health complaints and medical attendance was investigated using the chi-squared test.

In a second step, multivariate binary logistic regression analysis was performed, including the influencing factors which were identified in the first step of analysis.

All statistical tests and bar charts were performed using SPSS 23 under application of 0.05 statistical significance level (two-sided).

## RESULTS

### Demographic Information

**Fig 1:**
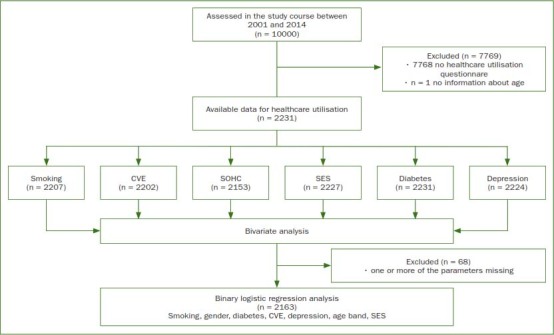
Diagram visualising the performance of data analysis; CVE: cardiovascular events (comprising heart attack and stroke), SOHC = subjective oral health complaints; SES = socioeconomic status.

From 2011 to 2014, 10,000 adults in the age between 18 and 79 (mainly > = 40) years were included in the LIFE-Adult-Study (Table 1, Fig 1). A total of 2231 participants were interviewed considering medical attendance and could be included in the analysis of healthcare utilisation behaviour in the present investigation. Table 1 gives an overview about demographic information and the prevalence of influencing factors within the different age groups.

**Table 1:** Description of the study sample

Age groups[years]	Net sample size n = 2231		Smoking	Diabetes	CVE	Depression	Low SES
	Females	Males					
18–34	44 (48.4%)	47 (51.6%)	36 (39.6%)	0 (0%)	0 (0%)	4 (4.4%)	25 (28.4%)
35–44	145 (47.2%)	162 (52.8%)	99 (32.6%)	4 (1.3%)	1 (0.3%)	28 (9.2%)	37 (12.1%)
45–54	308 (46.5%)	355 (53.5%)	205 (31.2%)	33 (5.0%)	12 (1.8%)	88 (13.3%)	100 (15.1%)
55–64	302 (47.6%)	332 (52.4%)	131 (21.0%)	73 (11.5%)	25 (4.0%)	93 (14.7%)	100 (15.8%)
65–74	189 (41.1%)	271 (58.9%)	42 (9.2%)	74 (16.1%)	26 (5.8%)	35 (7.7%)	51 (11.1%)
75–79	56 (73.7%)	20 (26.3%)	3 (4.1%)	15 (19.7%)	7 (9.3%)	6 (7.9%)	8 (10.5%)
Total	1105 (49.5%)	1126 (50.5%)	516 (23.4%)	199 (8.9%)	71 (3.2%)	254 (11.4%)	321 (14.4%)

### Dental and Medical Attendance

Considering dental attendance, statistically significant differences were found in association to different age groups and gender (Table 2). A total of 23.1% from the 18-to-34-years age group visited a doctor, but not the dentist (Table 3). 7.7% of them did not attend medical service at all (Table 3). With increasing age, a higher percentage of participants utilised both a dentist and at least one kind of physician (Table 3). Female subjects showed more attendance to medical service than men; 17.1% of male participants did not visit the dentist, compared with 11.1% of the female subjects (p <0.001; Tables 2, 3).

**Table 2:** Characterisation of study participants considering dental attendance in dependence of influencing factors

Influencing factor	age (mean ± SD)	Study subjects with information about dental attendance n = 2231		p value
		Visit to the dentist 85.8% (n = 1908)	No visit to the dentist 14.2% (n = 316)	
		56.0 ± 11.3	54.3 ± 13.1	<0.001^*^
% of age group (n)	18–34 (n = 89)	69.2 (63)	30.8 (28)	0.001†
	35–44 (n = 244)	86.0 (264)	14.0 (43)	
	45–54 (n = 617)	87.2 (578)	12.8 (85)	
	55–64 (n = 622)	86.3 (547)	13.7 (87)	
	65–74 (n = 582)	86.3 (397)	13.7 (63)	
	75–79 (n = 282)	86.8 (66)	13.2 (10)	
Gender	% of females (n)	88.9 (982)	11.1 (123)	<0.001†
	% of males (n)	82.9 (933)	17.1 (193)	
Smoking	% yes (n) n = 517	81.8 (423)	18.2 (94)	p = 0.003†
	% no (n) n = 1694	87.1 (1476)	12.9 (218)	
CVE	% (n) n = 71	80.3 (57)	19.7 (14)	n.s. (p >0.05)†
SES	% low (n) n = 322	78.0 (251)	22.0 (71)	p = 0.000022†
	% moderate to high (n) n = 1909	87.2 (1664)	12.8 (245)	
DM	% yes (n) n = 199	88.4 (176)	11.6 (23)	n.s. (p >0.05)†
	% no (n) n = 2012	85.6 (1722)	14.4 (290)	
Depression	% yes (n) n = 254	82.3 (209)	17.7 (45)	n.s. (p >0.05)†
	% no (n) n = 1970	86.3 (1700)	13.7 (217)	
Medical attendance (overall)	% yes (n) n = 2095	86.3 (1809)	13.7 (109)	p = 0.007
	% no (n) n = 140	77.9 (109)	22.1 (31)	
GP n = 2224	% yes (n) n = 2095	86.0 (1642)	79.7 (251)	p = 0.005
	% no (n) n = 140	14.0 (267)	20.3 (64)	
Medical specialists n = 2086	% yes (n) n = 1948	93.9 (1692)	89.8 (256)	p = 0.014
	% no (n) n = 138	6.1 (109)	10.2 (29)	

SD: Standard deviation; † chi-square test.

**Table 3:** Characterisation of study participants considering medical attendance in dependence of influencing factors

Influencing factor		Study subjects with information about medical attendance n = 2231				p value
		Dentist 10.0% (n = 223)	Doctor 11.5% (n = 256)	Doctor and dentist 75.8% (n = 1692)	No dentist, no doctor 2.7% (n = 60)	
Age		50.6 ± 10.2	54.9 ± 13.2	56.4 ± 11.3	56.8 ± 11.3	<0.001^*^
% of age group (n)	18–34	15.4 (14)	23.1 (21)	53.8 (49)	7.7 (7)	<0.001†
	35–44	17.3 (53)	11.4 (35)	68.7 (211)	2.6 (8)	
	45–54	11.8 (78)	9.8 (65)	75.4 (500)	3.0 (20)	
	55–64	9.5 (60)	11.0 (70)	76.8 (487)	2.7 (17)	
	65–74	3.9 (18)	12.0 (55)	82.4 (379)	1.7 (8)	
	75–79	0 (0)	13.2 (10)	86.8 (66)	0 (0)	
Gender	% of females (n)	4.9 (54)	10.1 (112)	84.0 (928)	1.0 (11)	<0.001†
	% of males (n)	15.0 (169)	12.8 (144)	67.9 (764)	4.4 (49)	
Smoking	% yes (n) n = 517	7.0 (36)	15.5 (80)	74.9 (387)	2.7 (14)	p <0.00012†
	% non (n) n = 1694	4.3 (72)	11.9 (201)	82.9 (1404)	1.0 (17)	
CVE	% (n) n = 71	0 (0)	14 (19.7)	57 (80.3)	0 (0)	p >0.05∫
SES	% low (n) n = 322	3.4 (11)	19.9 (64)	74.5 (240)	2.2 (7)	p = 0.00035†
	% moderate to high (n)n = 1909	5.1 (97)	11.6 (221)	82.1 (1567)	1.3 (24)	
DM	% yes (n) n = 199	0 (0)	11.6 (23)	88.4 (176)	0 (0)	p = 0.000089∫
	% no (n) n = 2012	5.3 (106)	12.9 (259)	80.3 (1616)	1.5 (31)	
Depression	% yes (n) n = 255	1.6 (4)	17.6 (45)	80.4 (205)	0.4 (1)	p = 0.002∫
	% no (n) n = 1973	5.3 (105)	12.2 (240)	81.0 (1598)	1.5 (30)	

*t-test; ∫ Fischer’s exact test; † chi-square test.

Regarding smoking, SES and medical status, statistically significant influence on dental attendance was found for smoking, SES and medical attendance in univariate analysis (p <0.05; Table 2). Considering overall medical attendance, smokers were more likely to visit exclusively the dentist or the physician or none of them, whereas the utilisation of both a dentist and a doctor was reduced, compared to non-smokers (p <0.001, Table 3).

Participants of low SES visited the dentist less frequently compared to subjects of moderate to high SES (p <0.0001, Table 2). Regarding medical attendance, a higher selective attendance of the physician but much lower attendance of the dentist could be observed in cases of low SES (p <0.001, Table 3).

Persons who reported dental attendance also showed significantly more often medical attendance, taking into account GP attendance as well as medical specialists (Table 2, p <0.01).

Medical and dental attendance differed significantly in participants who stated to suffer from depression: they visited more often exclusively the physician and showed less dental attendance (p <0.01, Table 3).

**Fig 2:**
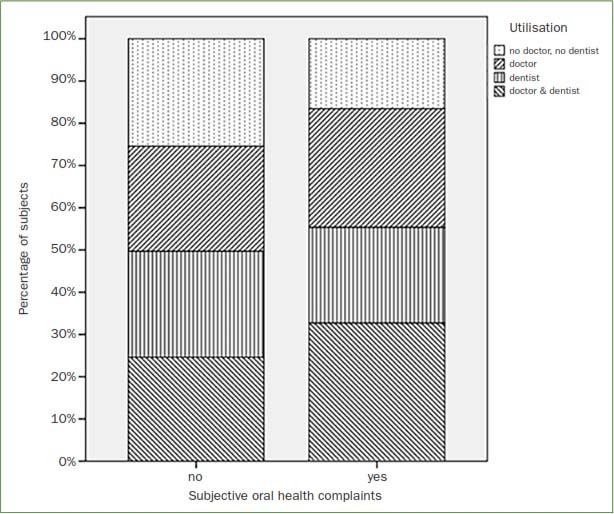
Grouped bar chart for dental and medical utilisation behaviour in participants with and without subjective oral health complaints (given in percentages).

The subcohort without oral health complaints contained a higher percentage of persons who utilised neither the doctor nor the dentist, compared to the subcohort with oral health complaints (p >0.05, Fig 2).

### Predictors of Unfavourable Dental Attendance

Binary logistic regression analysis was performed to assess factors influencing dental attendance behaviour and identify possible risk indicators for unfavourable, because irregular, utilisation behaviour. The findings are reporting the use of dental services. Therefore, the dependent variable was ‘visit to the dentist during the last 12 months’ (yes or no). In multivariate analysis (Table 4), the covariates smoking (OR = 0.7, CI: 0.6–1.0) female gender (OR = 1.8, CI: 1.4–2.3), depression (OR = 0.6, CI: 0.4–0.9), low SES (OR = 0.6, CI= 0.4–0.8) and age of 35–44 (OR = 2.4, CI: 1.3–4.2) were statistically significant (p ≤ 0.01).

**Table 4:** Binary logistic regression model for analyzation of factors influencing dental attendance

	Risk factor	p	OR (exp(B)) to have had a dental visit during the last 12 months compared with reference category (95% Confidence interval [CI])
Smoking	No†	0.045	
	Yes		0.75 (0.6, 1.0)
Gender	Male†	<0.001	
	Female		1.8 (1.4, 2.3)
Diabetes	No†	n.s.	
	Yes		1.5 (0.9, 2.4)
Cardiovascular event	No†	n.s.	
	Yes		0.711 (0.4, 1.3)
Depression	No†	0.01	
	Yes		0.6 (0.4, 0.9)
Age group	18–34†		
	35–44	0.003	2.4 (1.3, 4.2)
	45–54	0.412	1.2 (0.8, 1.8)
	55–64	0.585	0.9 (0.7, 1.3)
	65–74	0.628	0.9 (0.6, 1.3)
	75–79	0.461	1.3 (0.6, 3.0)
SES	High to moderate†	<0.001	
	Low		0.6 (0.4, 0.8)

†Reference category.

## DISCUSSION

The study aimed at considering dental utilisation behaviour in relation to medical attendance as well as demographic factors, general health status and subjective oral health complaints in a German population aged 18–79 years. The results showed differences in dental attendance depending on age, gender, SES, smoking status and depression. Furthermore, an association between dental and medical attendance was found.

A fundamental strength of the present study is, that this is, to our knowledge, the first study investigating utilisation behaviour towards dentists compared to GPs as well as medical specialists and taking into account general medical conditions. Moreover, the LIFE-Adult-Study is population-based and stands out due to its group size of 10,000 included participants. However, the questionnaire dealing with utilisation of dental and medical services was applied in only 2231 study participants. Since the questionnaire was applied randomly and in all age groups, the cohort considered in the present investigation, stays representative. Furthermore, because of the local restriction in participant recruitment in Greater Leipzig, the study is to be regarded as representative of central, but not of the whole of Germany. Due to the longitudinal design, it is possible to invite the participants for follow-up investigation in the future, which is an enormous strength in itself.^14^ The present investigation has a number of limitations. Firstly, the analysed data were obtained by questionnaire survey, exclusively. In the run-up to the study, participants were requested to bring a list of their medications and diseases to the appointment. Medical and cardiac histories were taken in interview style by a trained physician, limiting mistakes due to misunderstandings to a minimum. However, the information given by the participants considering their histories were not approved by their treating doctors or their health insurances. Secondly, the statements of the 2231 subjects with evaluable results considering dental and medical attendance behaviour could not be verified in the individual case. Thirdly, no information about the reasons (routine or problem-orientated) for dental or medical utilisation was obtained in this study. Therefore, the interpretation is limited without being able to differentiate between regular or symptom-related utilisation behaviour.

Regarding the results of the current study in the context of the recent literature, few studies exist concerning the utilisation behaviour of medical and dental service in industrialised countries. For Germany, the DMS V revealed that 84.2% of study participants (n = 959) in the age between 35 and 44 and 89.8% (n = 913) of the 65–74-year-old group utilised the dentist during the last 12 months.^11^ This could be confirmed by the present study which found comparable results with 86.0% and 86.3% for the age groups of 35–44 and 65–74, respectively. Both of the studies, the DMS V and the present study, revealed a higher percentage of the younger seniors to attend the dentist regularly compared to the younger adults. Considering medical utilisation behaviour, similar trends, found in the current study, are reported by the study ‘Gesundheit in Deutschland Aktuell’ (GEDA) of the Robert Koch Institute; almost 9 out of 10 adults attended outpatient physical care during 1 year.^13^ Chronic diseases, eg, DM and CVE, were shown to be associated with a higher number of visits to a physician.^20^ This confirms our findings for participants with DM and CVE. All of them sought medical care during the last 12 months (Table 3). However, to our best knowledge, no data exist considering dental utilisation behaviour in German patients suffering from chronic diseases, like DM, CVE and depression. The literature-based discussion of the results, therefore, is only possible to a limited extend. Tomar and Lester showed that US adults with DM were less likely to visit the dentist during the preceding 12 months than those without diabetes; a great disparity was observed depending on SES and racial or ethnic groups.^24^ This result was not confirmed in the current study. However, differences in the health insurance system between Germany and the US, differences in ethnicities and much lower number of DM patients in the current study might limit comparability of the results.

Results of a recent study performed by Csikar et al show that smokers were twice as likely to attend the dentist symptomatically compared to non-smokers.^5^ Taking into account that symptomatic dental attendance can be regarded as an irregular utilisation behaviour, the results of the current study show a similar trend of the dental attendance of smokers.

For DM and CVE, interactions with oral health conditions, especially periodontal disease, have been sufficiently demonstrated.^18^,^22^ The same interrelation was found for depression and low SES. Some authors already suspect an association between periodontitis and depression,^6^,^10^ but a recently published review underlined the necessity of further research.^1^Regarding SES, different studies found a relationship between unfavourable dental attendance and low SES,^7^,^11^,^17^ which was confirmed by the results of the current study as well.

## CONCLUSIONS

The present study reveals that patients who do not visit the dentist show irregular attendance of medical services (GP as well as medical specialists); risk factors for irregular and therefore unfavourable dental and medical attendance could be identified. Based upon the presented results, in the cases of low SES and depression, a possible influence of appropriate information by the GP or medical specialist is assumable and could be implemented quite easily. Especially in subjects aged 18 to 34 years, dental teams should be aware of an elevated risk for irregular dental attendance and pay increased attention not to lose these patients out of regular control. These aspects should be considered in patient management in dental as well as medical practices.
